# Genetic mapping of loci involved in oil tocopherol composition control in Russian sunflower (*Helianthus annuus* L.) lines

**DOI:** 10.1093/g3journal/jkac036

**Published:** 2022-02-12

**Authors:** Rim Gubaev, Stepan Boldyrev, Elena Martynova, Alina Chernova, Tatyana Kovalenko, Tatyana Peretyagina, Svetlana Goryunova, Denis Goryunov, Zhanna Mukhina, Cecile Ben, Laurent Gentzbittel, Philipp Khaitovich, Yakov Demurin

**Affiliations:** 1 Skolkovo Institute of Science and Technology, Moscow 121205, Russia; 2 LLC “Oil Gene”, Moscow 121205, Russia; 3 Pustovoit All-Russia Research Institute of Oil Crops, Krasnodar 350038, Russia; 4 Russian Potato Research Center, Kraskovo 140051, Russia; 5 Vavilov Institute of General Genetics, Russian Academy of Sciences, Moscow 119333, Russia; 6 Belozersky Institute of Physico-Chemical Biology, Lomonosov Moscow State University, Moscow 119992, Russia; 7 All-Russia Rice Research Institute, Krasnodar 350921, Russia

**Keywords:** oil oxidative stability, genotyping by sequencing, genetic linkage maps, QTL mapping, tocopherol double mutant, Russian germplasm

## Abstract

Tocopherols are antioxidants that preserve oil lipids against oxidation and serve as a natural source of vitamin E in the human diet. Compared with other major oilseeds like rapeseed and soybean, sunflower (*Helianthus annuus* L.) exhibits low phenotypic diversity of tocopherol composition, both in wild and cultivated accessions from germplasm collections. Two major mutations that alter tocopherol composition were identified in genetic collections, and several studies suggested additional loci controlling tocopherol composition, with their expression possibly depending on the genetic background. In the present study, we performed QTL mapping of tocopherol composition in two independent F2 crosses between lines with contrasting tocopherol composition from the Pustovoit All-Russia Research Institute of Oil Crops (VNIIMK) collection. We used genotyping-bysequencing (GBS) to construct single nucleotide polymorphism-based genetic maps, and performed QTL mapping using quantitative and qualitative encoding for phenotypic traits. Our results support the notion that the tocopherol composition in the assessed crosses is controlled by two loci. We additionally selected and validated two single nucleotide polymorphism markers for each cross which could be used for marker-assisted selection.

## Introduction

Tocopherols are plant-derived metabolites that play diverse roles in plant physiology from the abiotic stress responses to plant development (Falk and Munné-Bosch 2010). Tocopherols are also an essential part of human and animal diet and are commonly known as vitamin E. These compounds exist in 4 different forms, namely α-, β-, γ-, and δ-tocopherols, which differ in the number and location of methyl groups in the chromanol ring. Notably, vitamin E activity decreases in the row from α- to γ-tocopherol, while in vitro antioxidant properties, on the contrary, increase (Demurin et al. 1996; Fernández-Martínez et al. 2007). In sunflower, low natural diversity of tocopherol composition is observed with the typical tocopherol form produced in sunflower being α-tocopherol (Demurin 1993; Demurin et al. 1996; Hass et al. 2006). It should be noted that changes in tocopherol composition may significantly affect sunflower oil properties. For example, increased content of γ- and/or δ-tocopherols positively affect oxidative stability of sunflower oil, especially in combination with altered oleic acid, thus improving its characteristic as a frying oil. So, directing sunflower breeding programs to alter γ- and/or δ-tocopherol content allows developing inbred lines and hybrids suitable for frying oil production (Skorić et al. 2008; Warner et al. 2008; Márquez-Ruiz et al. 2014).

The biosynthesis of different tocopherols in sunflower is controlled by three key enzymes. The first one is 2-methyl-6-phytyl-1,4-benzoquinone (MPBQ) methyltransferase (MPBQ-MT) which converts MPBQ to 3,2-dimethyl-6-phytyl-1,4-benzoquinone (DMPBQ). At this stage, it is determined whether further biosynthesis steps will lead to the accumulation of α- and γ- or β- and δ-tocopherols. At the next step, MPBQ or DMPBQ are transformed to δ- and γ-tocopherols, respectively, by tocopherol cyclase (TC). The last step in the tocopherol biosynthesis pathway is controlled by γ-tocopherol methyltransferase (γ-TMT), which converts γ- and δ-tocopherols to α- and β-tocopherols, respectively (Fritsche et al. 2017). In sunflower, the tocopherol biosynthesis pathway normally leads to the accumulation of α-tocopherol through the DMPBQ => γ-tocopherol (γ-T) => α-tocopherol (α-T) branch (Hass et al. 2006).

Since altered tocopherol composition is of high interest for plant breeders, genetic loci associated with tocopherol composition control were mapped in most important oilseed crops including rapeseed, soybean, and sunflower (Haddadi et al. 2012; Wang et al. 2012; Sui et al. 2020). For sunflower, two major genes encoding MPBQ-MT and γ-TMT have been described, mutations in which may lead to altered tocopherol composition. The *Tph1* (also called *m*) locus corresponds to the gene encoding MPBQ-MT (MT-1) (Tang et al. 2006). The impaired form of *Tph1* was identified in the LG15 line from the VNIIMK collection (Demurin 1993) and in the T589 line from the Institute of Sustainable Agriculture (CSIC) (Velasco et al. 2004). Both lines demonstrate increased β-tocopherol content. The second locus *Tph2* (also called *g*) corresponded to the γ-TMT gene (Hass et al. 2006) and was identified in the LG17 line from VNIIMK and in the T2100 line from CISC (Velasco et al. 2004). Both lines show the increased γ-tocopherol phenotype. Genetic analysis of *Tph1* from LG15 and T589 as well as *Tph2* from LG17 and T2100 demonstrated the same phenotypic effects regardless of which genetic collection the line originated (Demurin et al. 2004; Vera-Ruiz et al. 2005). Importantly, *Tph1* was mapped to chromosome 1, while *Tph2* was mapped to chromosome 8 (Velasco et al. 2004; Vera-Ruiz et al. 2005), and these loci were shown to be independent (Demurin 1993; Demurin et al. 2004). However, it remains unknown if causative mutations in *Tph1* and *Tph2* genes are the same in VNIIMK and CSIC lines.

Apart from *Tph1* and *Tph2*, additional loci have been discussed to alter the tocopherol composition in sunflower. A locus named *d* was shown to interact epistatically with *m* (*Tph1*) and *g* (*Tph2*) altering tocopherol composition (Hass et al. 2006). The *d* locus contains the gene coding for MPBQ-MT transferase (MT-2) that is paralogous to the one encoded by *m* (*Tph1*) locus (Tang et al. 2006). Additionally, several loci have been reported to alter specifically γ-tocopherol composition in sunflower plants carrying the mutant *tph2* alleles. In particular, the IAST-1 and nmsT2100 lines that carry altered *tph2* alleles were shown to demonstrate stable high γ-tocopherol profiles. However, the mutant *tph2* allele from IAST-1 was shown to be affected by additional modifying genes in a different way compared to the nmsT2100 line (García-Moreno et al. 2012). Above-mentioned studies suggest that tocopherol composition in sunflower is on the one hand associated with the two independent loci with major effects that carry mutations resulting in impaired MPBQ-MT and γ-TMT enzymes, and on the other hand, could be affected by additional loci carrying modifying genes with minor effects. Furthermore, it has been hypothesized that tocopherol composition is also affected by the genetic background of the studied lines (Demurin et al. 1996) which implies that genetic markers for tocopherol composition may be specific for certain plant material.

In the previous studies moderate density genetic maps for sunflower has been obtained (García-Moreno et al. 2006; Hass et al. 2006; Tang et al. 2006; Vera-Ruiz et al. 2006; García-Moreno et al. 2012) based on SSR and indel markers, which possibly did not allow to take into account all recombination events. In the present study, we apply the genotyping-by-sequencing (GBS) approach (Elshire et al. 2011) that allows assembly of high-density SNP-based linkage maps to perform fine mapping of the loci associated with the altered tocopherol composition. Here, we have used two independent mapping populations derived from the genetically contrasting lines obtained from the VNIIMK collection that are currently used in the breeding process. The work was carried out with the aim of scanning for potential genetic markers for the future introduction of marker-associated selection (MAS) approaches with regard to tocopherol composition in sunflower.

## Materials and methods

### Plant material used in the study

The plant material included genetically contrasting double-mutant lines VK195 (*tph1/tph1*; *tph2/tph2*) and VK876 (*tph1/tph1*; *tph2/tph2*) expressing γ- and δ-tocopherol phenotypes (∼50% of γ- and ∼50% δ-tocopherols). As a wild-type VK303 (*Tph1/Tph1*; *Tph2/Tph2*) and VK101 (*Tph1/Tph1*; *Tph2/Tph2*) lines expressing high α-tocopherol phenotype (100% of α-tocopherol). All lines were developed at the VNIIMK institute. Elite lines VK101 and VK303 are used to produce a simple interlinear middle-early sunflower hybrid “Typhoon” registered at VNIIMK (Trembak et al. 2018). Lines VK876 and VK195 are used to produce “Oxy” hybrid which oil demonstrate high content of oleic acid and γ- and δ-tocopherol forms that increase oxidative stability (Demurin et al. 2016). Notably earlier, it was demonstrated that VK876 and VK195 possess high expressivity of *tph1* and *tph2* mutation (Demurin et al. 2006) and thus are of high interest to be introduced in elite lines VK101 and VK303 in order to obtain valuable hybrids with oil resistant to oxidation.

Fertile lines VK195 and VK876 were hand emasculated to prevent self-pollination and were used as females. Fertile VK101 and VK303 were used as male lines to produce the pollen. The crosses were performed in the field (Krasnodar) in 2015. Individual F1 plants were selfed using isolators to produce the F2 progeny (F2 seeds) in the field (Krasnodar) in 2018. Seeds were obtained from a single inflorescence that was randomly selected from a plot. Seeds of parental plants used for phenotyping and genotyping procedures were used from the VNIIMK collection.

### Phenotyping procedures

The analyses were performed for F2 seeds randomly sampled per F1 sunflower head, for a total of 142 F2 seeds for cross VK195xVK303 and 144 F2 seeds for the cross VK876xVK101. Seeds were harvested at the physiological maturity stage (R-9) according to the sunflower growth stages classification (Schneiter and Miller 1981). For phenotyping, a half-seed technique previously used to analyze the oil-related traits in sunflower (Demurin et al. 2004; Tahmasebi Enferadi et al. 2004; Vera-Ruiz et al. 2005) was applied: a single seed was cut in half with a razor, and the first part containing the embryo was left for germination for subsequent genotyping, while the second part was used for phenotyping.

The composition of tocopherols in half seeds was determined using thin layer chromatography. A portion of the excised seed cotyledons (10–30 mg) was placed in a tube, adding 20 mg of ascorbic acid, crushed with a glass rod, then 0.5 ml of 2 N KOH solution in 96% ethanol was added. The tube was kept in a water bath at 80°C for 20 min. After saponification, 0.5 ml of water and 1 ml of hexane were added. After addition of the hexane, the tube was shaken and left on the table until phases separated, then the upper hexane layer with a fraction of unsaponifiable matter, including tocopherols, was taken. Residual hexane was evaporated, and the rest of the solution was applied to a silica gel plate for thin layer chromatography using a mechanical applicator Sorbfil. A mixture of hexane and diethyl ether in a ratio of 4:1 was used as a solvent. For the detection of tocopherols, the plate was sprayed with a freshly prepared mixture of 0.1% ferric chloride (FeCl_3_) and 0.25% α,α′-dipyridyl in absolute ethanol (Emmery-Engel’s reagent), taken by volume in a ratio of 1:1. The stained plates were scanned and the relative composition in tocopherols forms was quantified by measuring the relative intensity of the color of tocopherol forms (in %) using a Sorbfil videodensitometer (software version 1.5.0). Parental plants were phenotyped in at least 7 biological replicates.

Using that data on relative content of each of 4 tocopherol forms, plants were described as having one of the 4 tocopherol-composition-specific phenotypic classes: α (>60% of α-, 0%–30% of β-, 0%–30% of γ-, 0%–10% of δ-tocopherol), α/β (from 15% to 70% of α and from 30% to 85% of β-, 0% for γ-, and δ-tocopherol), γ (10%–65% of α-, 0% of β-, 35%–100% of γ-, and 0%–21% of δ-tocopherol), γ/δ (8%–40% of α-, 0%–25% of β-, 25%–84% of γ-, and 8%–50% of δ-tocopherol). The phenotypic segregation ratio was tested using χ^2^ goodness-of-fit test. The results on the ratio between the tocopherol class are summarized in Supplementary Table 1.

### Genotyping procedures

To perform genotyping, the half-seeds of the sunflower lines left after cotyledon separation were germinated in rolls of filter paper, after which DNA was extracted from the cotyledon leaves using NucleoSpin Plant II kit (Macherey-Nagel) according to the manufacturer’s manual. GBS library was prepared according to a modified protocol (Elshire et al. 2011) as described previously (Goryunov et al. 2019; Chernova et al. 2021). GBS library sequencing was performed in Illumina HiSeq 4000 with single-end reads with a length of 150 bp. Parental plants were genotyped in at least 7 biological replicates (Supplementary Table 2). Raw sequence data are available on NCBI SRA under the project number PRJNA742188. Plant samples and corresponding barcodes are summarized in Supplementary Table 2.

### SNP calling, genotype imputation, and genetic map construction

To collect data on single-nucleotide polymorphisms (SNPs), TASSEL-GBS (Glaubitz et al. 2014) pipeline v2 was used. To carry out read mapping, bowtie2 (Langmead and Salzberg 2012) with –very-sensitive parameter was used. Sunflower XRQ2.0 genome assembly (https://www.heliagene.org/HanXRQr2.0-SUNRISE/) was used as a reference (Badouin et al. 2017). To determine the genetic diversity among parental lines, the SNPs were collected for each parental line’s replicate. The principal component analysis was performed using PLINK software version 1.9 with default parameters (Purcell et al. 2007). For genetic map construction, reads from each parental library were merged before SNP calling. Only SNPs that were homozygous within each parent and polymorphic between the parents were selected for genetic map construction. SNPs that were genotyped in less than 20% of individuals, as well as SNPs that demonstrated less than 10% or more than 90% of heterozygosity level among individuals were removed as described previously before imputation procedure (Fragoso et al. 2016). Imputation was performed using default parameters using LB-impute pipeline (Fragoso et al. 2016). For genetic map construction, SNPs genotypes were converted into the A, B, and H format where A corresponds to maternal allele, B corresponds to the paternal one, and H to heterozygous state using custom R script (https://github.com/RimGubaev/vcf_to_qtl). Before genetic map construction, SNPs with a significant segregation distortion (Bonferroni-corrected χ^2^ goodness-of-fit test *P*-value < 0.05) as well as ones that were genotyped in less than 80% of individuals were filtered out. Individuals that were genotyped by less than 80% of SNPs were also removed.

For genetic map construction, R/qtl software (Broman et al. 2003) was used. Location data on physical chromosomes was used as reference to preorder the loci. For final marker ordering within physical chromosomes, the Kosambi mapping function was applied. Additional filtering for SNPs within each chromosome was performed using the droponemarker() function; markers with LOD scores equal or above −20 were discarded. The Pearson correlation coefficient and the corresponding significance level between physical and genetic distances was estimated using cor() and cor.test() functions in the R base package.

### QTL mapping

For QTL mapping procedures, two approaches were used: method using quantitative trait data and method using qualitative trait data. For quantitative mapping of quantitative traits, the proportion of each of the tocopherol classes was considered as an independent observation. For those traits, the interval mapping approach based on Haley–Knott regression for nonnormally distributed traits was applied using the scanone() function implemented in the r/qtl package. For QTL mapping of *Tph1* loci based on qualitative trait data, the phenotype of plants that belong to the α (*Tph1*/*_*; *Tph2*/*_*) and γ (*Tph1*/*_*; *tph2*/*tph2*) classes was set to 1, while the α/β (*tph1*/*tph1*; *Tph2*/*_*) and γ/δ (*tph1*/*tph1*; *tph2*/*tph2*) classes were set to 0. For QTL mapping of the *Tph2* mutation based on qualitative trait data, the phenotype of plants that belong to the α (*Tph1*/*_*; *Tph2_*) and α/β (*tph1*/*tph1*; *Tph2*/*_*) classes was set to 1, while the β (*Tph1*/*_*; *tph2*/*tph2*) and γ/δ (*tph1*/*tph1*; *tph2*/*tph2*) classes was set to 0. The corresponding phenotypes of plants that did not belong to any of the classes were set to NA (Supplementary Table 1). To scan for loci associated with qualitative trait variation induced by the *tph1* and *tph2* mutations, the interval mapping approach based on Haley–Knott regression for binary traits implemented in the r/qtl’s scanone() function was used.

The significance threshold for both of the methods was set using permutation analysis for the logarithm of odds (LOD) carried out with 1,000 iterations to find significant loci. The LOD value corresponding to the 99 percentile of the permuted LOD values distribution was set as the significance threshold. To calculate the proportion of variance explained, analysis of variance (ANOVA) was applied within the R statistical package. LOD intervals were estimated using the lodint() function in r/qtl and 1.5-LOD confidence intervals were calculated with the expansion of the interval to the closest flanking marker.

### SNP variant validation

To validate the allelic states of polymorphic SNPs obtained with GBS, SNPs from within the 1.5-LOD confidential interval calculated for *Tph1-* and *Tph2-*associated SNPs were selected. To design primers for SNP validation sequences surrounding the markers were extracted using bedtools v2.27.1 (Quinlan and Hall 2010), so as to have 500 bp both upstream and downstream of the target polymorphic site (Supplementary fasta file). Primers were designed using the Primer3 plus online tool (Untergasser et al. 2012), primer self-complimentary and possible dimer formation was checked using the OligoCalc tool (Kibbe 2007), primer specificity was verified with the aid of the PrimerBlast software (Ye et al. 2012) and using direct BLAST with the sunflower XRQ2.0 genome assembly as a reference implemented in the PlantEnsemble genome browser.

The criteria for selecting SNPs for validation were to have among the highest LOD score among the identified SNPs for the corresponding population (VK195xVK303/VK876xVK101) and gene (*Tph1*/*Tph2*) combination and among the highest percentage of correctly predicted phenotypes among the plants for which genotypes were known based on GBS sequencing. The ultimate criterion for SNP selection among those showing the highest LOD score and correctly predicted phenotype percentage was their location in a unique genome region. It should be noted that it appeared challenging to design primers for PCR amplification in the case of almost all SNPs primarily selected for validation. The major concern was mispriming leading to a high amount of possible nonspecific amplification products due to high homology levels between the target regions and other locations in sunflower genome as revealed by PrimerBlast and Ensemble-Blast validation. For this reason, only those polymorphic SNPs which were located in the unique regions thus allowing to design a unique pair of primers were chosen for subsequent validation steps. For marker validation, we selected 34 F2 plants from the mapping population including 8 plants carrying the parental genotype, 8 carrying the maternal genotype, and 8 heterozygous plants. Additionally, we used 5 female and 5 male parent plants which were used to obtain the F2 populations. Plants were selected based on the GBS results. For marker validation, direct Sanger sequencing of the amplicons obtained using the corresponding primer pairs was used. Sanger sequencing results were analyzed using the Ugene software (Okonechnikov et al. 2012). Raw sequencing data are attached as Supplementary files.

Genomic DNA previously isolated for GBS sequencing was used for PCR amplification. PCR reaction mixture contained 50 ng of template DNA, 2.5 μl of SmartTaq 10× buffer with 1.5 mM MgCl_2_, 0.25 μM each of the forward and reverse primers, 0.20 mM dNTPs, and 1.25 U of *Taq* polymerase. PCR reagents were all purchased from Dialat (Moscow, Russia). DNA amplification conditions were as follows: 95°C for 3 min; 35 cycles of 95°C for 1 min; primer-specific temperature for 30 s, and 72°C for 30 s. The reaction was terminated after the extension at 72°C for 5 min. The resulting PCR products were separated on 2.5% agarose gel, the amplicon bands were excised and purified using the Qiagen MinElute Gel Extraction kit (Qiagen, United States) prior to sequencing. Sanger sequencing was performed by Evrogen (Russia).

To test the concordance of the allelic states, we compared the genotypes of the markers that were obtained using GBS with the ones obtained by Sanger sequencing and calculated the proportion of the matching genotypes. Sequence analysis of the resulting amplicons confirmed the consistency of polymorphisms for the SNP variants for the parental lines and F2 populations.

## Results

### Plant Phenotyping

Each of the parental lines used for crosses was phenotyped in at least 7 replicates, and the mean values were used. The maternal lines carrying *tph1* and *tph2* mutations showed a clear γ/δ phenotype with the almost equal γ-/δ-tocopherol proportions, while the paternal lines demonstrated the wild-type α phenotype (Table 1).

**Table 1.** Description of the parental lines.

Line name	Genotype	Tocopherol class	Mean proportion of α-tocopherol, %	Mean proportion of β- tocopherol, %	Mean proportion of γ-tocopherol, %	Mean proportion of δ-tocopherol, %
BK195	*tph1*/*tph1* *tph2*/*tph2*	γ/δ	0	0	51.7 ± 5.41	48.3 ± 5.41
BK303	*Tph1*/*Tph1* *Tph2*/*Tph2*	α	100	0	0	0
BK876	*tph1*/*tph1* *tph2*/*tph2*	γ/δ	0	0	52.9 ± 8.71	47.1 ± 8.71
BK101	*Tph1*/*Tph1* *Tph2*/*Tph2*	α	100	0	0	0

The mean values are displayed along with the standard deviation of raw data.

**Fig. 1.**
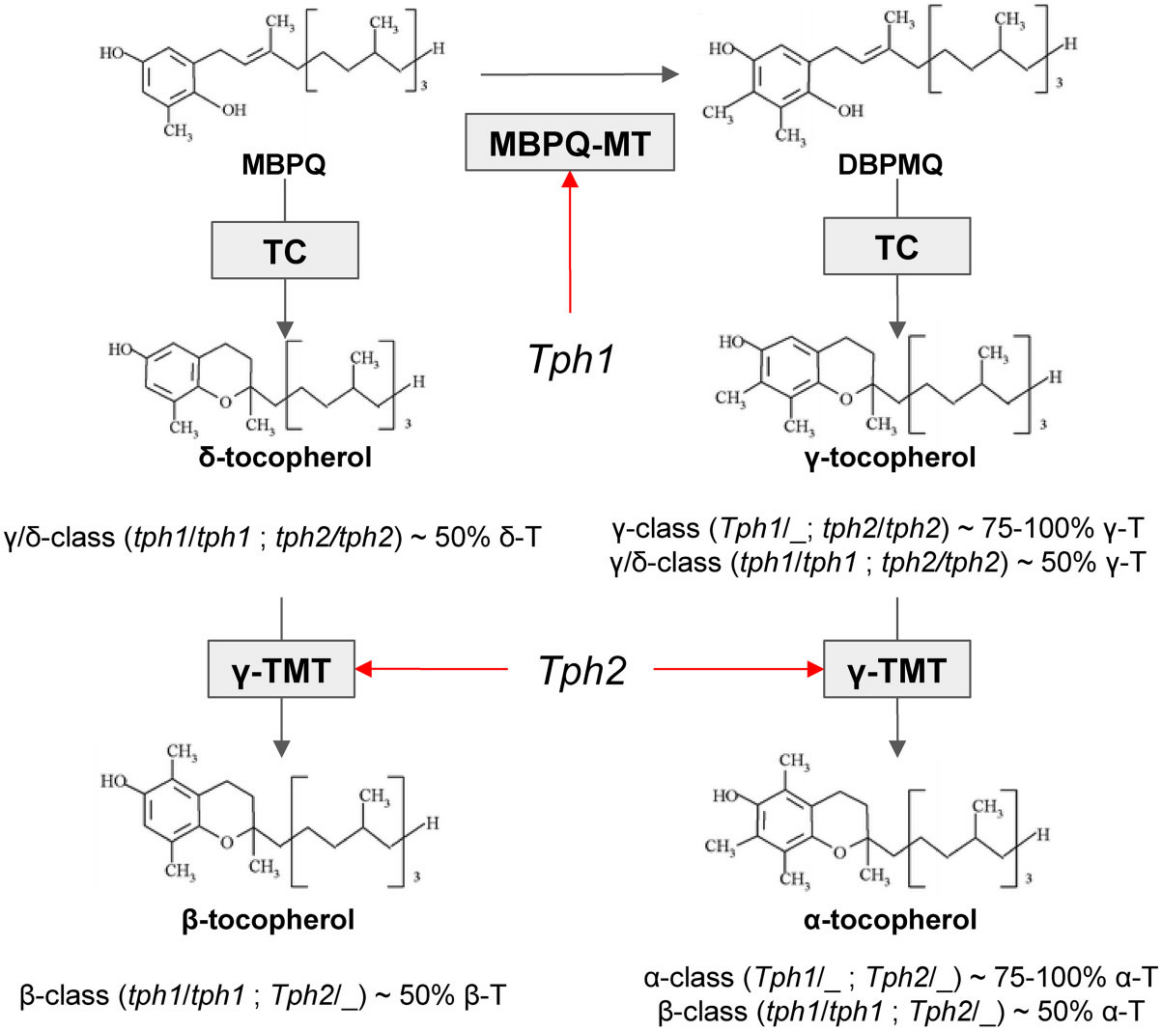
Tocopherol biosynthesis scheme with corresponding phenotype classes. Names of the metabolites are indicated in bold: 2-methyl-6-phytyl-1,4-benzoquinone—MPBQ; 3,2-dimethyl-6-phytyl-1,4-benzoquinone—DMPBQ. Gray arrows correspond to the reactions catalyzed by the enzymes indicated in the gray squares: MPBQ methyltransferase—MPBQ-MT; tocopherol cyclase—TC; γ-tocopherol methyltransferase—γ-TMT. Red arrows indicate enzymes encoded by *Tph1* and *Tph2*. The biosynthesis pathway scheme was adapted from Lushchak and Semchuk (2012).

As a result of phenotyping procedures for progeny, 142 F2 seeds from the VK195xVK303 cross and 144 F2 seeds from the VK876xVK101 cross were characterized for their tocopherol composition (Supplementary Table 1). The classification was performed based on the putative allelic states of the genes which in turn affects the tocopherol composition (Fig. 1).

**Fig. 2.**
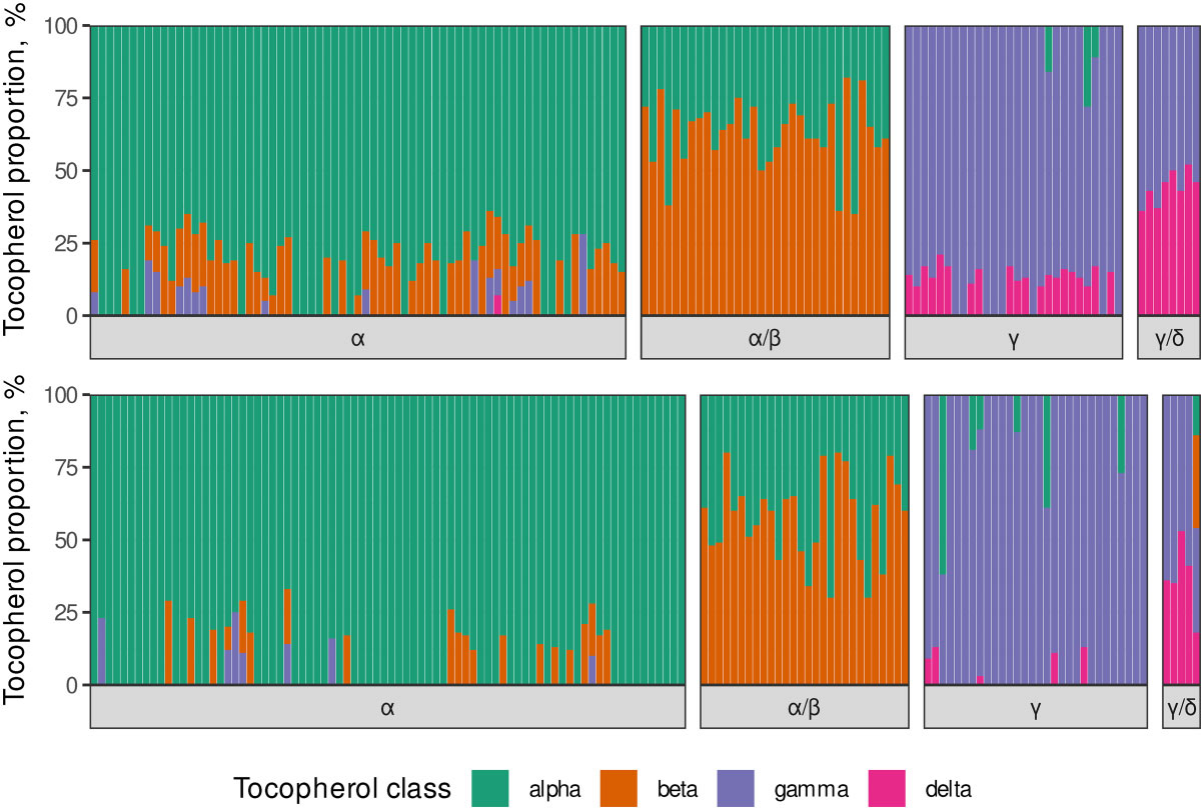
Relative content of each of the tocopherol classes among the genotyped individuals in the F2 progeny. Each bar corresponds to a single F2 seed. Each colored bar shows the proportion of each of the four tocopherol classes. Facets show the distribution of phenotyped F2 seeds across the α-, α/β-, γ-, and γ/δ-tocopherol phenotypic classes. Upper panel corresponds to the VK195xVK303 cross. Lower panel corresponds to the VK876xVK101 cross.

Each plant was assigned into one of the four tocopherol classes defined by the ratios of tocopherol forms (Fig. 2). In total, out of 142 phenotyped F2 seeds from the VK195xVK303 cross and out of 144 F2 seeds from the VK876xVK101 cross, 137 (96.4%) and 143 (99.3%) were classified into the known phenotypic classes, respectively (Fig. 2, Table 2, and Supplementary Table 1).

**Table 2.** Number of plants assigned to one of the 4 tocopherol classes for each of the crosses.

Cross	α-class	α/β-class	γ-class	γ/δ-class	*P*-values for χ^2^ goodness-of-fit test
VK195xVK303	69	32	28	8	0.4505
VK876xVK101	80	28	30	5	0.5382

Only 1 F2 seed from VK195xVK303 and 5 F2 seeds from VK876xVK101 were not assigned to any specific phenotypic class due to their tocopherol ratios deviating from the specified classes (Supplementary Table 1). According to the χ^2^ goodness-of-fit test for the classified progeny, phenotype classes matched the 9:3:3:1 distribution (Table 2 and Fig. 2) with high confidence (*P*-values of 0.4505 and 0.5382 for VK195xVK303 and VK876xVK101, respectively).

### Genotyping, SNP calling, and genetic map construction

**Fig. 3.**
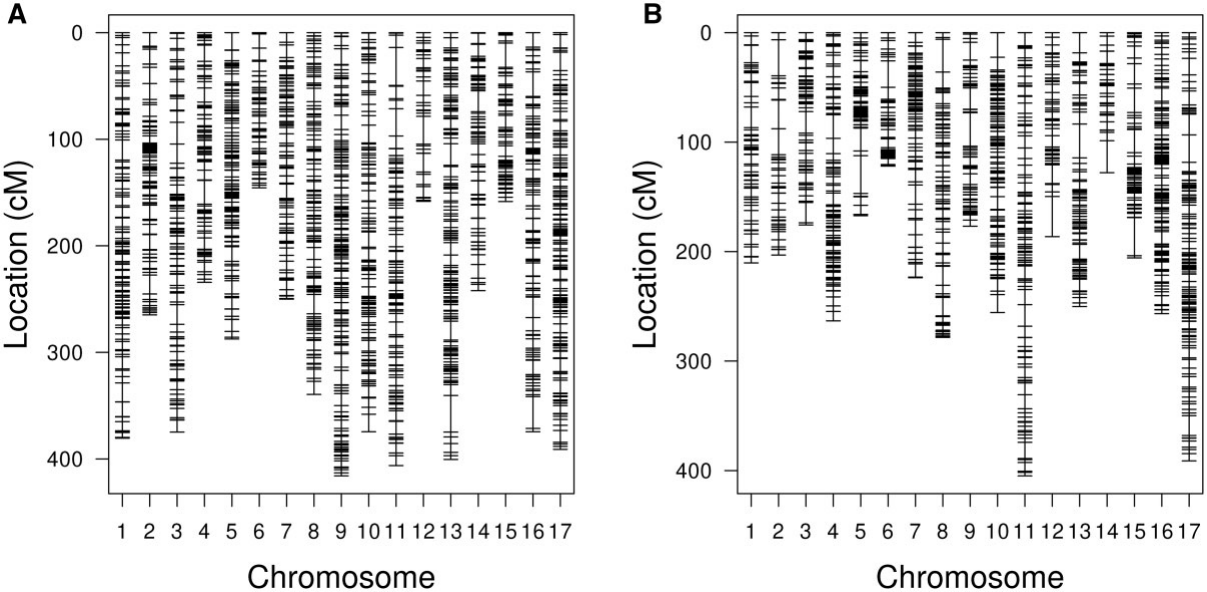
SNP-based genetic linkage maps for the VK195xVK303 a) and VK876xVK101 b) crosses. Linkage maps for VK195xVK303 and VK876xVK101 crosses are based on 3,200 and 2,571 SNPs, respectively.

To perform genotyping of parental and progeny plants, we used a GBS approach (Elshire et al. 2011). Seven plants of each parental line, as well as 142 and 144 plantlets were genotyped for the VK195xVK303 and VK876xVK101 crosses, respectively (Supplementary Table 2). Using the Tassel GBS-V2 pipeline, 425,213 and 499,063 raw SNPs were obtained for VK195xVK303 and VK876xVK101, respectively. After additional filtering, 7,028 and 5,876 SNPs remained for further analysis for the VK195xVK303 and VK876xVK101 crosses, respectively. Using the LB-impute approach, the proportion of missing genotypes was decreased from 0.22 to 0.1 and from 0.2 to 0.09 for the VK195xVK303 and VK876xVK101 crosses, respectively. Using imputed and filtered genotypes, genetic maps for both crosses were constructed (Fig. 3).

The genetic map for the VK195xVK303 cross included 3,200 markers and had a total distance of 5,197.7 cM. The average intermarker distance was 1.63 cM and the maximum spacing was 35.5 cM. The correlation between the genetic and physical distances was significant (*P*-value < 1.223024e-19) and ranged from 0.62 to 0.97 across the linkage groups (Table 3).

**Table 3.** Summary for the constructed genetic maps.

Chromosome	Cross	Number of markers	Map length (cM)	Average spacing (cM)	Maximum spacing (cM)	Pearson correlation coefficient	*P*-value for Pearson correlation coefficient
1	VK195xVK303	189	380.54	2.02	18.87	0.9	6.01E-68
2		188	264.79	1.42	17.82	0.72	3.1E-31
3		163	374.72	2.31	20.43	0.86	7.8E-49
4		136	234.29	1.74	22.41	0.85	1.18E-39
5		246	287.46	1.17	16.34	0.82	3.18E-62
6		95	145.51	1.55	13.32	0.88	1.52E-32
7		173	250.03	1.45	13.48	0.62	1.22E-19
8		238	339.45	1.43	18.24	0.94	1.42E-109
9		315	415.84	1.32	14.74	0.97	1.15E-188
10		240	374.47	1.57	16.69	0.95	1.42E-119
11		194	406.14	2.1	35.49	0.62	2.14E-22
12		57	158.27	2.83	26.02	0.91	2.02E-22
13		216	400.4	1.86	34.37	0.93	4.96E-98
14		126	242.1	1.94	19.66	0.9	3.36E-46
15		127	158.39	1.26	22.72	0.91	1.79E-48
16		187	374.41	2.01	29.86	0.79	1.8E-41
17		310	390.89	1.27	17.23	0.92	1.2E-126
Overall		3,200	5,197.71	1.63	35.49	—	—
1	VK876xVK101	98	210.29	2.17	18.77	0.9	1.7E-36
2		77	203.15	2.67	32.93	0.93	7.75E-34
3		118	175.66	1.5	19.68	0.9	8.98E-45
4		190	263.26	1.39	21.52	0.96	3.44E-106
5		138	167.13	1.22	34.49	0.89	2.48E-49
6		104	121.91	1.18	24.18	0.89	1.13E-36
7		215	223.92	1.05	17.5	0.48	5.21E-14
8		184	278.34	1.52	29.87	0.92	1.92E-75
9		104	176.82	1.72	30.57	0.95	5.43E-54
10		225	255.63	1.14	22.49	0.89	1.58E-79
11		137	404.86	2.98	19.73	0.9	1.25E-49
12		100	186.25	1.88	36.6	0.81	3.54E-24
13		165	250.15	1.53	30.8	0.96	9.5E-90
14		75	127.86	1.73	29.11	0.9	1.52E-27
15		105	205.8	1.98	34.89	0.94	7.57E-50
16		236	256.59	1.09	12.07	0.87	2.24E-73
17		300	391.19	1.31	24.98	0.89	2.8E-102
overall		2,571	3,898.81	1.53	36.6	—	—

The genetic map for the VK876xVK101 cross included 2,571 markers and had a total distance of 3,898.8 cM. The average intermarker distance was 1.53 cM and the maximum spacing 36.6 cM (Table 3). The Pearson correlation between the genetic and physical distances was significant with the highest *P*-value 5.205962e-14. Pearson correlation coefficients ranged from 0.48 to 0.96 for chromosomes from VK876xVK101 cross (Table 3).

### QTL mapping of tocopherol composition

**Fig. 4.**
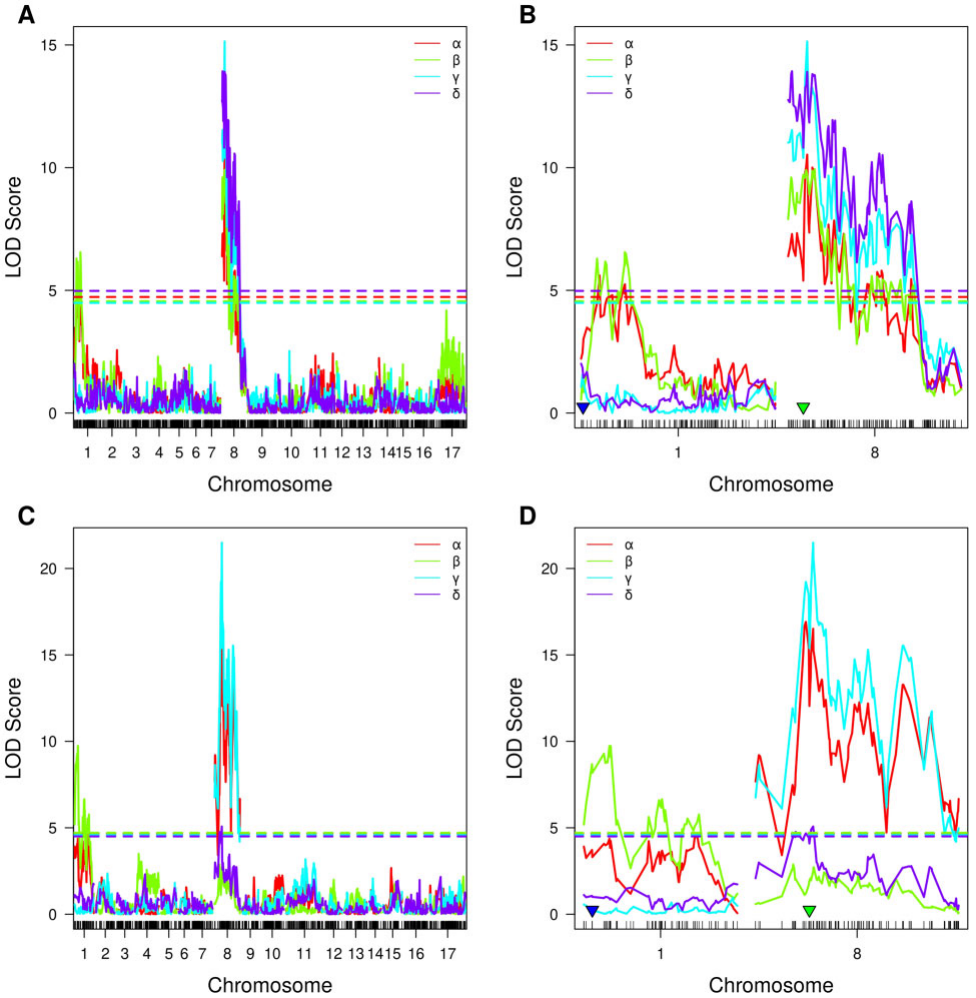
Likelihood curve of the LOD score for α-, β-, γ-, and δ-tocopherol content for the VK195xVK303 a and b) and VK876xVK101 c and d) populations obtained by using interval mapping approach. Dashed lines correspond to the permutation threshold. The color of lines corresponds to the tocopherol type. a and c) The mapping results for all chromosomes. b and d) The mapping results for chromosomes carrying significant markers. Blue and green triangles indicate markers that are most closely located to the *Tph1* and *Tph2* loci, respectively, based on the physical map data.

After the phenotype data were collected and genetic maps constructed, we performed the QTL mapping of the relative content of each of the tocopherol classes (Supplementary Table 3). As a result, we found loci significantly associated with all four tocopherol classes in both crosses (Fig. 4). Loci associated with α- and β-tocopherol content are located on chromosome 1 and 8 in both crosses, while the loci associated with γ- and δ-tocopherol content are located on chromosome 8 only in the VK195xVK303 cross. For the VK876xVK101 cross, it was shown that β tocopherol content is associated with the loci located on chromosome 1, while α, γ, and δ content were associated with the loci located on chromosome 8.

To estimate the impact of the loci that were identified by interval mapping, we selected markers from the 1.5-LOD interval for the most significant marker for each of the phenotypes for each of the crosses and calculated the proportion of phenotypic variance explained by the selected markers (Supplementary Table 4). For the VK195xVK303 cross, the maximum percentage of phenotypic variance explained by the markers located on the eighth chromosome for α, β, γ, and δ were 43.77%, 38.89%, 59.89%, and 39.69%, respectively. For α-, and β-tocopherols, the loci located on the first chromosome additionally explained up to 21.98% and 38.14% of phenotypic variance. For the VK876xVK101 cross, the maximum percentage of phenotypic variance explained by the markers located on the eighth chromosome for α, γ, and δ was 71.09%, 85.74%, and 9.83%, respectively. For β-tocopherols, markers located on chromosome 1 explained the maximum of 44.44% of phenotypic variance.

### QTL mapping of *Tph1* and *Tph2*

**Fig. 5.**
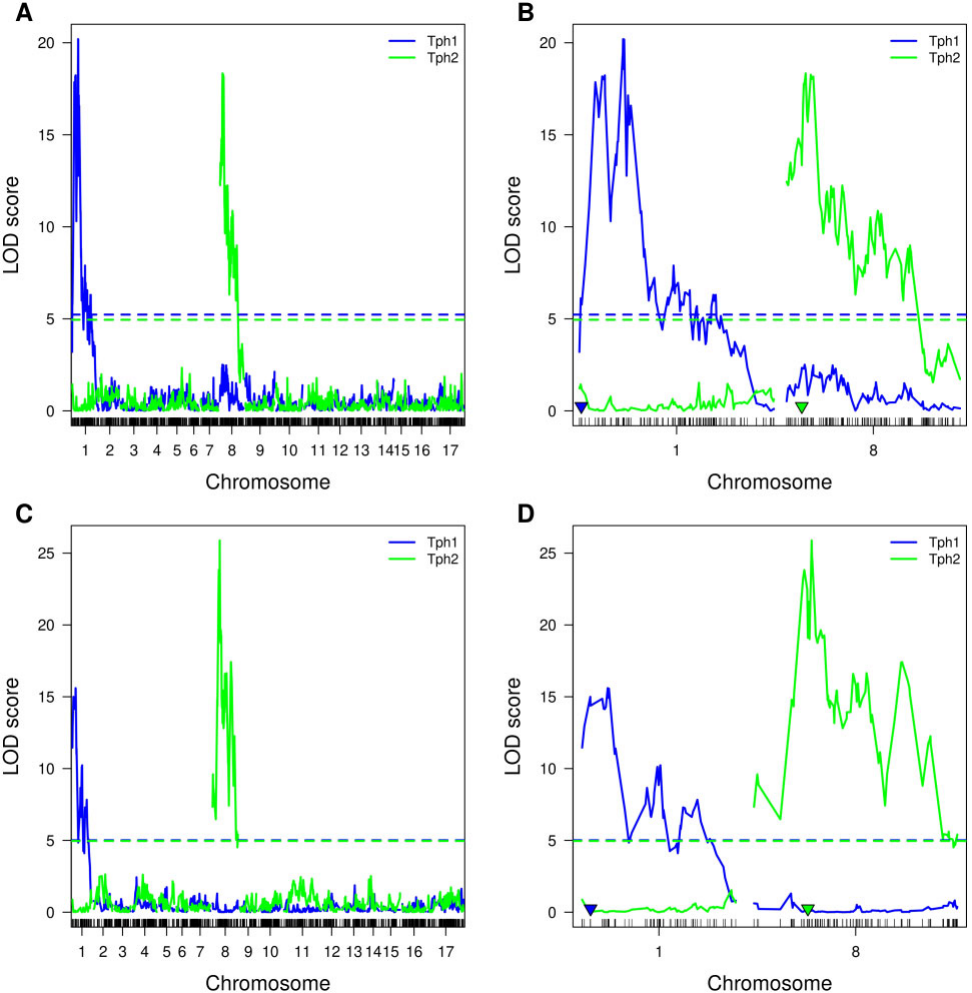
Likelihood curve of the LOD score for *Tph1* and *Tph2* for the VK195xVK303 a and b) and VK876xVK101 c and d) populations. Dashed lines correspond to permutation results. Interval mapping results for *Tph1* and *Tph2* are indicated with the corresponding colors. a and c) The mapping results for all chromosomes. b and d) The mapping results for chromosomes carrying significant markers. Blue and green triangles indicate markers that are most closely located to the *Tph1* and *Tph2* loci, respectively, based on the physical map data.

In addition to the quantitative mapping of the relative content of α-, β-, γ-, and δ-tocopherols, we performed the qualitative mapping of the *Tph1* and *Tph2* genes. In the present study, dominant/recessive states of these genes define the belonging of the plant phenotype (tocopherol composition) into one of the 4 tocopherol classes (Figs. 1 and 2 and Supplementary Table 1) and thus serve as a major factor affecting the tocopherol composition. As a result of *Tph1* and *Tph2* mapping, 2 genetic loci associated with the corresponding phenotypes were identified on chromosomes 1 and 8, respectively, for both mapping populations (Fig. 5 andSupplementary Table 5). For the VK195xVK303 cross, the 1.5-LOD confidence interval for *Tph1* spanned from 77.77 to 93.35 cM with the maximum LOD score of 20.19. For *Tph2*, the 1.5-LOD confidence interval spanned from 32.12 to 64.69 cM with a maximum LOD score of 18.33.

For the VK876xVK101 cross, the highest LOD peak indicating association with *Tph1* was located from 3.05 to 44.54 cM demonstrating maximum LOD value of 15.6. For *Tph2*, the region with the highest LOD scores spanned from 75.98 to 83.57cM with respective maximum LOD values equal to 24.65.

Notably, markers that were located most closely to the *Tph1* based on physical distance data demonstrated significant LOD scores of 6.11 (marker S1_9228105) for the VK195xVK303 cross (Fig. 5b and Supplementary Table 3) and 14.37 (marker S1_4480460) for the VK876xVK101 cross (Fig. 5d and Supplementary Table 3). For *Tph2*, the corresponding LOD values of the closest significant markers were 13.34 (S8_21613153) and 19.10 (S8_21536716) for the VK195xVK303 (Fig. 5b) and VK876xVK101 (Fig. 5d) crosses, respectively. According to the information on physical distances, the closest significant markers were located at the distance of 0.5 and 5.3 megabases to the actual *Tph1* (NCBI Gene ID: 110937001) for the crosses VK195xVK303 and VK876xVK101, respectively, this gene was previously shown to encode MPBQ-MT controlling β-tocopherol biosynthesis (Tang et al. 2006). As in the case with *Tph1*, markers significantly associated with *Tph2* were also closely located (i.e. 0.34 and 0.42 Mb, respectively, for K195xVK303 and VK876xVK101) to the *Tph2* locus that carries 2 genes (NCBI Gene IDs: 110872346 and 110872347) encoding γ-TMT1 and γ-TMT2, involved in γ- and δ-tocopherol accumulation in sunflower (Hass et al. 2006).

### SNP marker validation

As the aim of the study was to find genetic markers associated with tocopherol composition, we selected and tested markers for *Tph1* and *Tph2* for each of two populations, since these genes determine the tocopherol composition for the analyzed crosses. The main criteria for selection was first the presence of the marker in the 1.5-LOD confidence interval (Supplementary Table 4), second the ability to construct primers for amplification and sequencing so that they would capture a unique DNA sequence. Based on the above-mentioned criteria, we selected markers S1_55196434 and S8_30578572 associated with *Tph1* and *Tph2* with respective LOD scores of 20.17 and 18.26 for VK195xVK303 cross, respectively. For VK876xVK101 cross, we selected S1_71748138 and S8_23941299 markers for *Tph1* and *Tph2* with respective LOD scores of 15.57 and 24.66. Then we constructed 4 pairs of primers in order to amplify 4 unique regions capturing genetic markers that underwent the subsequent Sanger sequencing (Table 4).

**Table 4.** Primers used to amplify and sequence unique regions containing SNPs of interest.

Primer name	Sequence 5′-3′	Tm used for amplification	Product size
S1_55196434_Tph1_VK195xVK303_F	ACATGGTTTCTTATCATTTGCAC	59.0 C	420
S1_55196434_Tph1_VK195xVK303_R	ACCGGATATTTGACAAAGTGC		
S8_30578572_Tph2_VK195xVK303_F	TGACTTACTTGGTCGAGCCG	60.5 C	416
S8_30578572_Tph2_VK195xVK303_R	GCACGTACCCGATTTCCTTG		
S1_71748138_Tph1_VK876xVK101_F	ATCCTCCACAACCCAACACG	60.0 C	459
S1_71748138_Tph1_VK876xVK101_R	GAAAGCATACTTTGGGCGACT		
S8_23941299_Tph2_VK876xVK101_F	TCTCGGATTACAGTGGTTCGA	59.5 C	287
S8_23941299_Tph2_VK876xVK101_R	GAAAACGATGGGGTTCTGG		

As a result, we sequenced and determined the allelic states of the four markers, namely, S1_55196434 and S8_30578572 for samples from VK195xVK303 population and S1_71748138 and S8_23941299 for samples from VK876xVK101 population (Supplementary Table 6). It has been demonstrated that the genotypes obtained using the GBS approach that were used for genetic map construction matched the resequenced genotypes from 85.29% to 94.12% (Table 5).

**Table 5.** Summary on SNP verification.

Marker	Chromosome	Position (cM)	Population	Gene	LOD score	The proportion of matching genotypes	Number matching genotypes
S1_55196434	1	88.71	VK195 X VK303	*Tph1*	20.18	94.12	32
S1_71748138	1	36.68	VK876 X VK101	*Tph1*	15.57	91.18	31
S8_23941299	8	79.34	VK876 X VK101	*Tph2*	24.66	85.29	29
S8_30578572	8	47.32	VK195 X VK303	*Tph2*	18.26	91.18	31

## Discussion

In the present study, we aimed to find genetic loci and corresponding markers responsible for tocopherol composition in perspective lines from Russian germplasm. To do so, we performed the phenotyping of two sunflower crosses derived from lines from VNIIMK collection. For the construction of SNP-based genetic maps, we applied a GBS approach. By means of QTL mapping, we identified new potential genetic markers and tested them using Sanger sequencing.

The phenotypic segregation in the two F2 populations obtained by crossing contrasting parental lines (Table 1 and Supplementary Fig. 1) statistically fits the four tocopherol classes within the 9:3:3:1 expected segregation ratio (Fig. 2 and Table 2). This is consistent with earlier studies focused on mapping the major effect loci *Tph1* and *Tph2.* Both loci present dominant/recessive allelic relationships and are unlinked (Vera-Ruiz et al. 2005; García-Moreno et al. 2006; 2006). Analyses of the phenotypic segregation in double-mutant lines from VNIIMK crossed with the wild-type ones (Demurin et al. 2006) confirmed this genetic model. However, several studies indicate that the phenotypic segregations of classical monohybridism (3:1) or dihybridism (9:3:3:1) for tocopherol composition may be distorted by the presence of additional genes (Hass et al. 2006; García-Moreno et al. 2012). To perform QTL mapping, we used GBS to construct an SNP-based genetic map. This approach made it possible to obtain a rather dense genetic map that we compared with the current genome assembly (Badouin et al. 2017). The first high-density SNP-based genetic map for sunflower was constructed using DNA microarrays (Bowers et al. 2012), followed by RAD-, SLAF-sequencing, and GBS-based maps, to decipher the genetic determinants of valuable traits in sunflower (Talukder et al. 2014; Celik et al. 2016; Zhou et al. 2018). These maps contain from 817 to 6,136 SNPs and span from 1,444 to 2,472 cM. In the present study, we report 2 maps (Fig. 3) of 3,899 and 5,198 cM in length carrying 2,571 and 3,200 markers, respectively (Table 3). Possible explanations for the fact that both genetic maps were almost twice as large as the previously reported ones may be as follows. First, we applied relatively relaxed filters for markers distorted in terms of expected segregation pattern of 1:2:1; second, the potential source of erroneous genotypes could be repetitive sequences that are highly present in sunflower genome (Badouin et al. 2017) and may lead to errors during fragment alignment (Kane et al. 2011; Treangen and Salzberg 2011). Notably, for other plant species, large chromosomes and genetic maps have also been reported previously. For sweet potato, which has 15 chromosomes, SNP-based genetic maps derived from RAD-seq data were up to 7,313.5 cM in length with the largest chromosome spanning 904.5 cM (Shirasawa et al. 2017). Relatively large chromosomes that exceeded 300 cM in length were also reported for wheat (Yang et al. 2018) and maize (Su et al. 2017; Wang et al. 2018). Importantly, the above-mentioned studies were also based on reduced-representation genotyping approaches. Apart from size and marker density properties, we additionally compared physical and genetic distances. In our case, Pearson correlation coefficient ranged from 0.48 to 0.97 (Table 3) which was higher than the 0.2 to 0.7 range previously reported for sunflower (Celik et al. 2016). This in turn evidences that the assembled genetic maps are in concordance with the physical ones.

Next we scanned for genetic associations for tocopherol composition. For both crosses, associations for β-tocopherol content were identified on chromosome 1 and associations for α-, γ-tocopherol content were identified on chromosome 8 (Fig. 4, b and d), which is in concordance with previously published results (García-Moreno et al. 2006; Tang et al. 2006; Vera-Ruiz et al. 2006). On the other hand, there were some discrepancies between the studied crosses; specifically, there was only a slight association in terms of the LOD score for δ-tocopherol in the VK876xVK101 population compared to the VK195xVK303 population. Additionally, there were no significant associations for α- and β-tocopherols on chromosomes 1 and 8, respectively, for the VK876xVK101 cross compared to VK195xVK303 cross. The differences in mapping results for the 2 studied populations could be affected by the different distributions of the relative content of tocopherol classes (Supplementary Fig. 1), notwithstanding the almost equal number of plants within each of the four phenotypic classes in both populations (Fig. 2 and Table 2). In addition, differences between populations were identified in the proportion of variance explained by the markers (Supplementary Table 4), namely, for the VK876xVK101 population, markers significantly associated with the α-, β-, and γ-tocopherols on average explained more variability compared to the VK195xVK303 population which could also be the result of the different relative distribution of tocopherol classes in the two populations.

Both populations demonstrated a relatively small portion of variance explained for β- and γ-tocopherols compared to the previous studies where β- and γ-tocopherol-associated markers explained up to 90% (Vera-Ruiz et al. 2006) and 97% of variance (García-Moreno et al. 2006), respectively. This may be explained by the fact that we analyzed a dihybridism situation, in which the expression of each of the tocopherol classes is affected by two loci simultaneously due to the dependency of tocopherol biosynthetic pathways on both enzymes (Fritsche et al. 2017). In contrast to the studies describing additional loci controlling tocopherol composition (Hass et al. 2006; Tang et al. 2006; García-Moreno et al. 2012), we did not found any markers significantly associated with the *d* mutation on chromosome 4 (Hass et al. 2006) carrying the paralog of *Tph1* contributing to α-tocopherol content. We also did not find any additional loci shown to be located on chromosomes 9, 14, and 16 which exert minor effects on γ-tocopherol content (García-Moreno et al. 2012). The lack of detection of additional loci could be the result of using relatively small F2 progeny samples or the effect of this loci could be masked with major effect loci located on chromosomes 1 and 8. This in fact makes these lines with high expressivity of *Tph1* and *Tph2* mutations associated with tocopherol composition (Demurin et al. 2004) convenient for use in breeding programs.

Thus on the next step, we applied a qualitative approach with the practical aim of finding potential genetic markers that could be used for marker-assisted selection. The progeny of the analyzed present study exhibited a clear 9:3:3:1 among four phenotype classes which allowed us to classify plants according to *Tph1-* and/or *Tph2-*associated phenotypes (Supplementary Table 1 and Fig. 2). As a result, *Tph1* was mapped to the upper end of chromosome 1 in both of the populations analyzed (Fig. 5, a and c). These results are in agreement with previous data where *Tph1* was also reported to be located on the upper end of the first chromosome in F2 crosses from different germplasms and likely different genetic backgrounds (Tang et al. 2006; Vera-Ruiz et al. 2006). For *Tph2*, we identified significantly associated markers located on chromosome 8 which is also in concordance with the results obtained for the CAS-12xIAST-540 F2 population (García-Moreno et al. 2006) and the R112 and LG24 F2 population (Hass et al. 2006). It should be noted that in the previous studies, *Tph1* and *Tph2* mapping was performed in a quantitative way i.e. the association between the genetic markers and β- and γ-tocopherol content, respectively was analyzed (García-Moreno et al. 2006; Hass et al. 2006; Tang et al. 2006; Vera-Ruiz et al. 2006). Notably, different statistical approaches were used to perform the mapping for *Tph1* and *Tph2* including single marker ANOVA (García-Moreno et al. 2006; Hass et al. 2006; Vera-Ruiz et al. 2006) and interval mapping (García-Moreno et al. 2006) as well as composite interval mapping (García-Moreno et al. 2012). Here, we have demonstrated that in our case, the qualitative approach based on interval mapping for traits with binary distribution was also suitable for mapping major effect loci and helped us to map *Tph1* and *Tph2* in both populations.

Since we found the SNPs strongly associated with *Tph1-* and *Tph2-*related phenotypes, we validated our GBS results by Sanger sequencing (Supplementary Table 6). The proportion of genotypes that matched between ones obtained by GBS and Sanger sequencing ranged from 85% to 94 %, making these SNPs prospective for marker-assisted selection. Notably, most of the mismatches were associated with the difference between the GBS-detected homozygous and heterozygous states detected by Sanger sequencing (Supplementary Table 6). This could be due to the fact that reduced representation sequencing approaches with relatively low coverage may result in heterozygote site undercoverage in populations with high heterozygosity (Swarts et al. 2014). And even the application of an imputation approach specifically developed for F2 and backcross populations (Fragoso et al. 2016) did not completely solve this problem.

Identified markers will be used for marker-assisted selection with the aim of altering tocopherol composition in the perspective lines, namely, shifting the overall tocopherol ratio from predominantly α-tocopherol to the higher content of β-, γ-, and δ-tocopherols. Since altered tocopherol composition depends on the presence of the recessive alleles of *Tph1* and/or *Tph2*, using molecular markers will significantly facilitate the accuracy and speed of progeny selection at the early developmental stages based on the genotype information. Notably, the identified markers could be used to introduce either the *tph1* allele associated with increased β-tocopherol content or *tph2* associated with high γ-tocopherol content as well as both recessive alleles of these genes leading to the accumulation of γ- and δ-tocopherols. In addition to the four markers verified in the present study, other SNPs from the 1.5-LOD intervals could also be utilized after the verification of corresponding GBS-detected genotypes, associated with *Tph1* and *Tph2*.

In conclusion, in the present study we have demonstrated that the tocopherol composition in four perspective sunflower lines from the VNIIMK collection is controlled by the two loci located on chromosomes 1 and 8. Which is in concordance with the previously published results (García-Moreno et al. 2006; Vera-Ruiz et al. 2006). We found and tested the genetic markers for *Tph1* and *Tph2* that could be used to distinguish between the tocopherol phenotypic classes and to trace these two loci in breeding programs aimed on the development of sunflower plants with altered tocopherol composition and increased oxidative stability of the oil.

## Data availability

The raw sequencing reads are available on NCBI SRA read archive under the project number PRJNA742188. Raw phenotype data are presented in Supplementary Table 1. Parental lines are available at the VNIIMK genetic collection upon request. Genetic maps available as Supplementary Material in csv format. The formatting of maps is suitable for the qtl R package. The genetic context of the markers that potentially could be used for MAS is available as Supplementary Material in fasta format. Raw data of Sanger sequencing is available as Supplementary Material in ab1 and seq formats in zip archive.

Supplemental material is available at *G3* online.

## Supplementary Material

jkac036_Supplemental_fasta

jkac036_Supplemental_Figure_1

jkac036_Supplemental_Material_1

jkac036_Supplemental_Material_2

jkac036_Supplementary_table_3

jkac036_Supplemental_Material_4

jkac036_Supplemental_Material_5

jkac036_Supplemental_Material_6

jkac036_Supplemental_Material_description
