# Supplementary figures and images for: Genetic mapping of loci involved in oil tocopherol composition control in Russian sunflower (Helianthus annuus L.) lines
Source: G3 (Bethesda). 2022 Feb 12;12(4):jkac036. doi: 10.1093/g3journal/jkac036 (PMC8982403; doi:10.1093/g3journal/jkac036)

**A**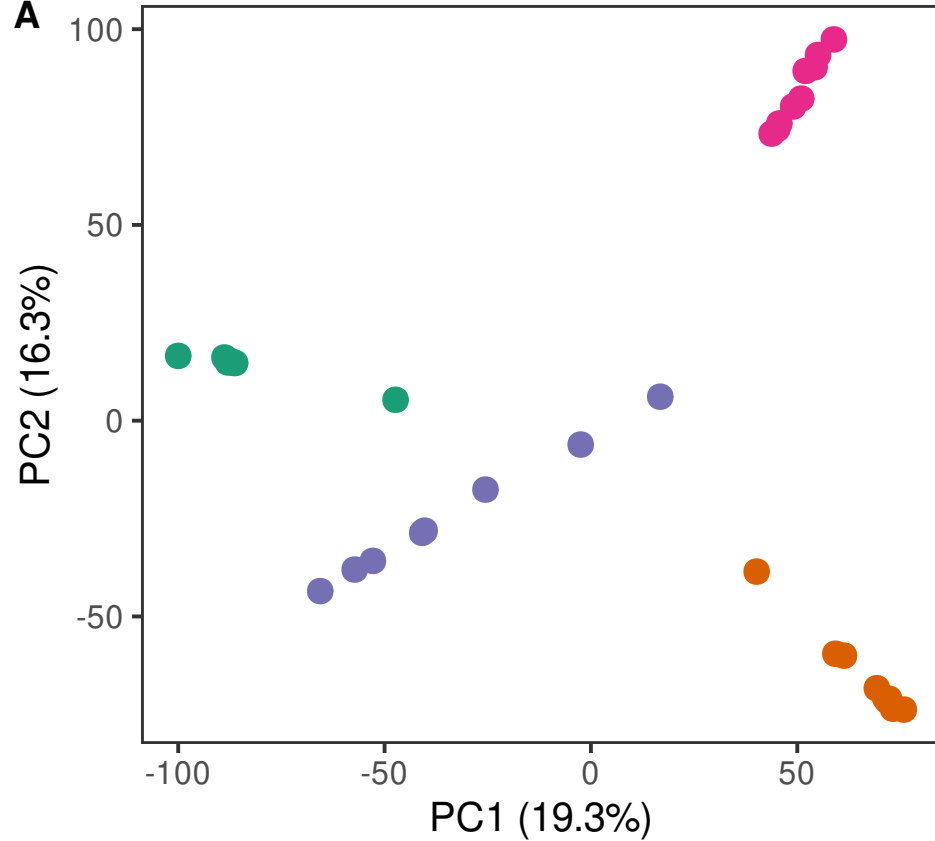**B**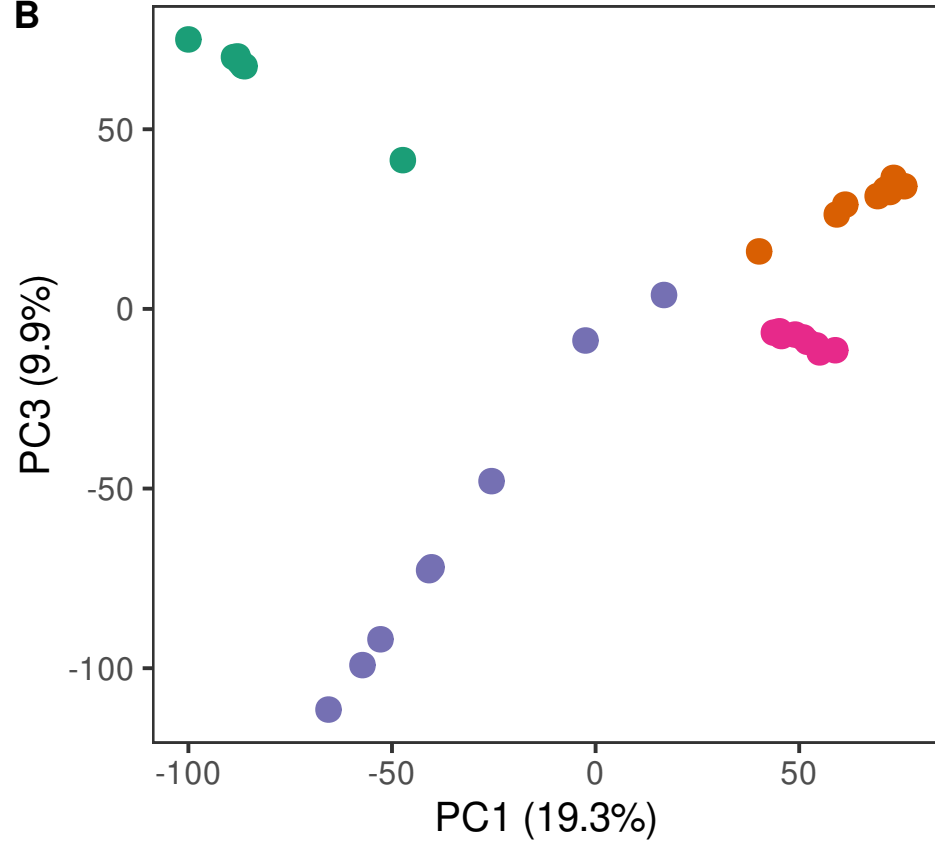

Line    VK101    VK195    VK303    VK876

Supplement: jkac036_Supplemental_Figure_1 [file jkac036_supplemental_figure_1.pdf]
