# Supplementary material for: Genetic mapping of loci involved in oil tocopherol composition control in Russian sunflower (Helianthus annuus L.) lines
Source: G3 (Bethesda). 2022 Feb 12;12(4):jkac036. doi: 10.1093/g3journal/jkac036 (PMC8982403; doi:10.1093/g3journal/jkac036)
Supplement: jkac036_Supplemental_Material_description [file jkac036_supplemental_material_description.docx]

**Supplementary files:**

Table S1 - Summary table on phenotype data.

Table S2 - List of the sequenced sunflower samples with corresponding replicate names and barcodes.

Table S3 - Summary table for QTL mapping results (LOD scores for each marker).Table S4 - ANOVA results for markers significantly associated with the relative content of α-, β-, γ-, and δ-tocopherols.

Table S5 - Markers associated with *Tph1* and *Tph2* linked phenotypes within 1.5-LOD intervals.

Table S6 - The comparison of GBS and Sanger sequencing detected genotypes.

Markers_sequence_tph1_tph2.fa - fasta files with 500bp upstream and downstream flanking sequence

Sequencing_results.zip - Sequencing results raw data

**Figure S1** - PCA plots depicting the population structure of studied parental lines. Color indicates the parental line.
